# Assessing the combined effect of multiple metal exposures on pregnancy and birth outcomes: Methodological insights in systematic review research

**DOI:** 10.1016/j.mex.2024.102558

**Published:** 2024-01-06

**Authors:** Ibrahim Issah, Mabel S. Duah, John Arko-Mensah, Serwaa A. Bawua, Thomas P. Agyekum, Julius N. Fobil

**Affiliations:** a West Africa Center for Global Environmental & Occupational Health, College of Health Sciences, University of Ghana, Legon, Accra; b Department of Surgery, Tamale Teaching Hospital, Tamale, Ghana; c Department of Biological, Environmental and Occupational Health, School of Public Health, College of Health Sciences, University of Ghana, Legon, Accra; d West African Center for Cell Biology of Infectious Pathogens, College of basic and Applied sciences, University of Ghana, Legon, Accra; e Department of Occupational and Environmental Health and Safety, School of Public Health, College of Health Sciences, Kwame Nkrumah University of Science and Technology, Kumasi 00233, Ghana

**Keywords:** Methods, Systematic review, Metals, Mixture analysis, Exposure, Birth outcomes, Methods for systematic review on metal mixture exposures and health outcomes: PECOS, PRISMA, and narrative synthesis approach.

## Abstract

This systematic review focused on pregnant women and aimed to evaluate how exposure to multiple metals impacts their pregnancy and birth outcomes. Previous research has predominantly focused on the effects of individual metal exposures on adverse birth outcomes. However, it is crucial to recognize that real-world scenarios often involve simultaneous exposure to multiple pollutants. Recent studies have emphasized the significance of considering exposure to mixtures of metals to gain a more comprehensive understanding of their collective health impacts. This article outlines the essential steps taken during the systematic review process, which involved synthesizing existing evidence and evaluating the strength and consistency of the relationship between metal mixtures and pregnancy and birth outcomes.

## Review Highlights

• PECOS framework to determine the inclusion criteria
• PRISMA strategy for study selection
• Narrative synthesis for study outcomes

## Graphical abstract

**Figure:**
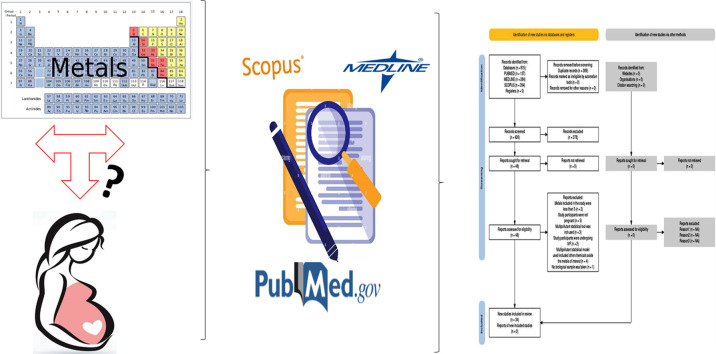

Specifications table

**Table:** 

Subject area:	Environmental Science
More specific subject area:	Exposure/Risk Assessment
Name of the reviewed methodology:	Methods for systematic review on metal mixture exposures and health outcomes: PECOS, PRISMA, and narrative synthesis approach
Keywords:	Methods
	Systematic review
	Metals
	Mixture analysis
	Exposure
	Birth outcomes
Resource availability:	1. http://dx.doi.org/10.1136/bmj.n71
	2. https://www.ohri.ca/programs/clinical_epidemiology/oxford.asp
Review question:	What is relationship between exposure to multiple metals and pregnancy and birth outcomes?

## Method details

### Background

Metals; such as lead (Pb), cadmium (Cd), and mercury (Hg), in the environment have been identified as potential risk factors for adverse effects on pregnant women and the developing fetus, including reproductive disorders, low birth weight, reduced birth length, reduced head and chest circumferences, and poor mental development [1]. While past reviews have mostly examined the effects of single metal exposure on adverse birth outcomes [2,3], this systematic review aimed to evaluate how exposure to multiple metals affects pregnancy and birth outcomes among adult pregnant women.

### Literature search

We developed a protocol that guided the conduct of our systematic review. The protocol involved the study's proposed title, research question, the scope and study design, and the search strategy. We applied the Participants/Population, Exposure, Comparator, Outcome, and Study Design (PECOS) framework to establish the inclusion criteria for the selection of eligible studies [4] (Table 1). Further exclusions included non-peer-reviewed publications and conference abstracts, and studies involving multiple pregnancies or prenatal conditions such as HIV infection, syphilis infection, preeclampsia, gestational diabetes, in vitro fertilization (IVF)-assisted pregnancies, or any other conditions linked to elevated risks of adverse pregnancy outcomes. The protocol was registered with PROSPERO and assigned an identification number (CRD42023422903).

**Table 1:** PECOS framework.

Criteria	Author application
Participants/Population	Adult pregnant women
Exposure	• Minimum of three different metals
	• Analyze metals individually at the level of human biological samples.
Comparator	Not applicable in this review
Outcome	Pregnancy or birth outcomes, including Birth weight (BW), Estimated fetal weight (EFW), Abdominal circumference (AC), Femur length (FL), Head circumference (HC), Biparietal diameter (BPD), Birth length (BL), Ponderal index (PI), Preterm birth (PTB), Small for gestational age (SGA), and Miscarriage
Study Design	• Primary investigations
	• Study designs: cohort, case-control, or cross-sectional designs
	• Utilize multipollutant statistical methodology, rather than the conventional regression approach.

In our review, we adhered to two distinct sets of guidelines to ensure a comprehensive and rigorous approach. The first set of guidelines was based on the updated version of the Preferred Reporting Items for Systematic Review and Meta-analysis (PRISMA 2020) checklist [5]. This checklist serves as a standardized framework for reporting systematic reviews and meta-analyses, ensuring transparency and completeness in our methodology. In addition, we incorporated the guidance on conducting systematic reviews and meta-analyses of observational studies of etiology (COSMOS-E) [6]. The inclusion of COSMOS-E was motivated by its specialized focus on observational studies, providing detailed guidance on various critical aspects of the review process. This comprehensive guide encompasses key considerations, such as formulating the research question, defining exposure and outcomes, evaluating the risk of bias, and conducting statistical analyses.

The decision to integrate COSMOS-E into our methodology was strategic, as it addressed specific challenges associated with systematic reviews of observational studies. By covering every step of the review process, from conceptualization to analysis, COSMOS-E complemented the PRISMA framework, enhancing the robustness and success of our review. This dual-guideline approach ensured a thorough and methodologically sound examination of the subject matter, aligning with the highest standards in systematic review methodology.

**Figure 1:**
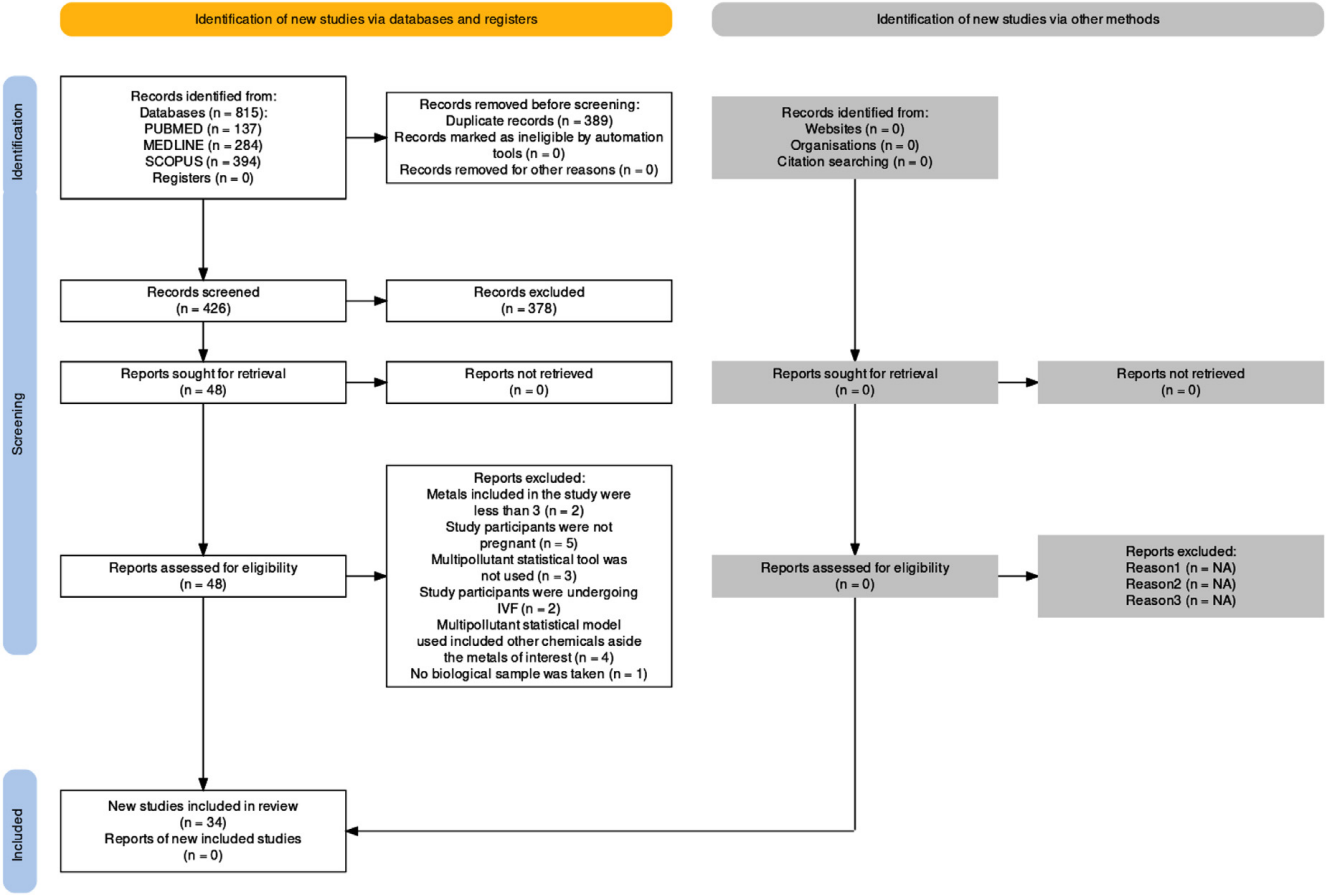
PRISMA flow diagram depicting the sequential process of selecting peer-reviewed articles for inclusion in the systematic review. Source: Haddaway, Page, Pritchard, and McGuinness [7]. *Note:* Three studies [[8], [9], [10]] met more than one exclusion criterion, contributing to the overall count of 17 studies excluded, even though there are 14 unique studies in the list.

We conducted an extensive search of PubMed, Medline, and Scopus using a combination of keywords and Boolean operators to identify articles on the association between heavy metals exposure and adverse pregnancy and birth outcomes. The search terms included various heavy metal names, birth outcome indicators, and statistical methods for assessing multiple pollutant effects (Table S1). We limited the search to peer-reviewed journal articles published in English from 1998 (when NIEHS first funded mixture research) to May 10, 2023. The same search strategy was applied to all three databases, and the search was initially performed on May 10, 2023, and later updated on July 31, 2023, using the same databases to ensure comprehensiveness. We updated the search to ensure that we captured the latest and most recently published articles relevant to the subject. By rerunning the search, we aimed to ensure the inclusion of any pertinent studies or findings that may have emerged subsequent to our initial search. This iterative approach to information retrieval enhanced the currency and comprehensiveness of our review. The literature search across three databases resulted in the identification of a total of 815 articles, of which 34 were identified as eligible studies (Fig. 1).

Because a high-quality study may still be at high risk of bias, we assessed both methodological quality and risk of bias assessment for each of the included study in our review. To assess the quality of the included studies, we employed two distinct qualitative tools tailored to the study designs under consideration. For cohort and case-control studies, we utilized the Newcastle‒Ottawa scale (NOS) [11] (Tables S2 and S3). In addition, for the evaluation of cross-sectional studies, we employed the AXIS Appraisal tool [12] (Table S4). While previous reviews have commonly employed the NOS for assessing the quality of cross-sectional studies [13,14], our review opted for the AXIS Appraisal tool. This decision was motivated by the specialized design of the AXIS tool, explicitly crafted for the nuanced evaluation of cross-sectional studies. By incorporating the AXIS Appraisal tool, we aimed to enhance the rigor of our review process, capitalizing on its focused assessment criteria, including reporting quality, design quality, and potential biases. This approach ensured a more thorough and specific evaluation of cross-sectional studies, contributing to the overall robustness and reliability of our review. Regarding the risk of bias assessment, we applied the adapted Office of Health Assessment and Translation (OHAT) approach developed by the National Institutes of Environmental Health Sciences National Toxicology Program [15].

Finally, we adopted a narrative synthesis approach to thoroughly examine each study included in our review. To enhance clarity and facilitate comprehension, we structured our presentation with subheadings that align with specific pregnancy and birth outcomes. This organizational framework was designed to provide a clear and coherent narrative, allowing readers to easily navigate and comprehend the findings related to each distinct aspect of pregnancy and birth.

## Literature analysis

The outcomes in this review were characterized as the results of conception and the subsequent pregnancy. We employed a narrative approach to organize the results into subheadings that correspond to specific pregnancy and birth outcomes. These subheadings are as follows:

1. **Birth Weight:** In this subsection, we collated and summarized the relevant studies that investigate the impact of metal mixtures exposure on birth weight outcomes.
2. **Preterm Birth:** Similarly, we created a dedicated subsection focusing on studies that explore the relationship between metal mixtures and preterm birth.
3. **Neonate Size:** This subsection contained a comprehensive discussion of studies addressing neonate size in the context of metal mixtures exposure. Various specific outcomes that represent neonate size were considered for discussion. These included estimated fetal weight (EFW), abdominal circumference (AC), femur length (FL), head circumference (HC), biparietal diameter (BPD), birth length (BL), and ponderal index (PI).
4. **Small for Gestational Age:** We dedicated a section to studies that investigate the prevalence of infants being born small for gestational age due to metal mixtures exposure.
5. **Others (Miscarriage and Placental Characteristics):** Lastly, we included a subsection to encompass findings related to miscarriage and placental characteristics, providing a holistic overview of our study outcomes

Each of these subheadings was designed to offer readers a focused and clear understanding of the specific pregnancy or birth outcome under investigation. We also incorporated summaries of the relationships between various metal elements and these outcomes within their respective subheadings.

**Figure 2:**
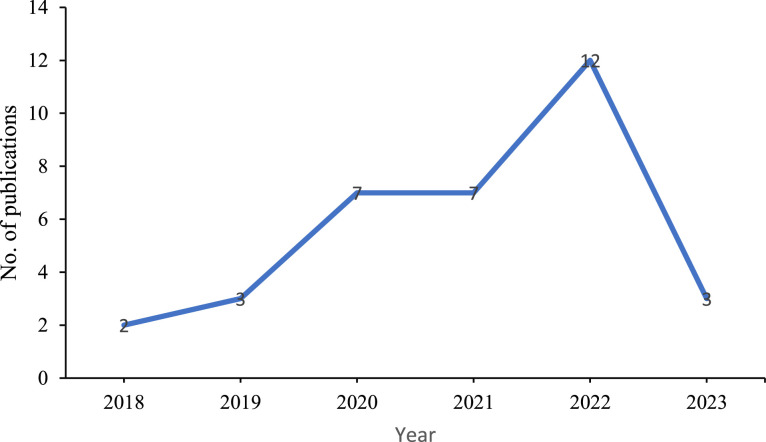
Annual scientific production.

**Figure 3:**
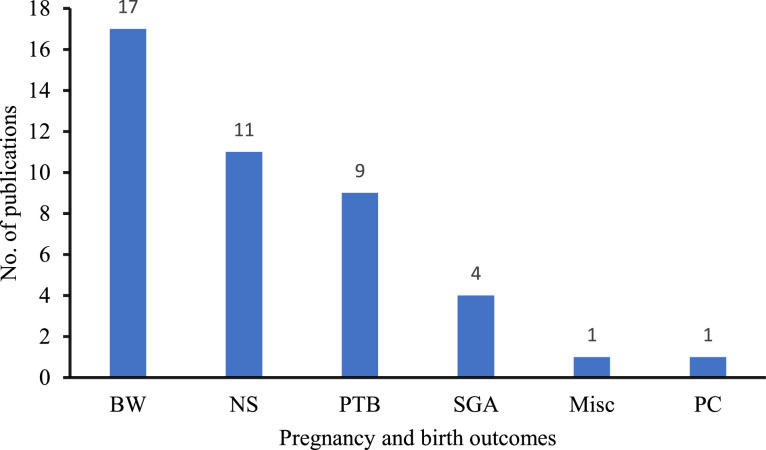
Summary of included studies that reported various pregnancy and birth outcomes. Abbreviations: BW – birth weight; NS – neonate size; PTB – preterm birth; SGA – small for gestational age; Misc – miscarriage; PC – placental characteristics. NB: Some studies reported more than one outcome.

In addition to presenting the results based on specific outcomes, the presentation of results was structured to ensure alignment with the geographical context. This approach provides a clearer and more accurate representation of the research studies conducted in specified regions. Finally, we incorporated visual representations to illustrate the growth in the field of metals mixture research over the past two decades (Fig. 2). Furthermore, we offered a summary showing the number of studies that reported specific outcomes (Fig. 3). This approach greatly enhanced the clarity and accessibility of the results, making them more intelligible to readers.

## Conclusion

This study underscores the important methodologies for conducting a systematic review of observational studies utilizing the PECOS, PRISMA, and narrative synthesis approach. The inclusion of diverse tools to assess methodological quality and risk of bias in individual studies adds a critical layer to the overall quality and reliability of the review's findings. These rigorous steps were fundamental in producing evidence that elucidates the collective impact of exposure to multiple metals on health. Our review outcomes illuminate the intricate interactions among different metals, encompassing additivity, synergism, and antagonism, and their influence on pregnancy and birth outcomes. We acknowledge the limitations of our study, particularly the absence of a meta-analysis for quantitative result pooling. Future reviews on mixture analysis should contemplate the incorporation of meta-analysis to offer a more comprehensive evaluation of the available evidence.

## Ethics statements

Not applicable

## Funding

This research did not receive any specific grant from funding agencies in the public, commercial, or not-for-profit sectors.

## CRediT authorship contribution statement

**Ibrahim Issah:** Conceptualization, Methodology, Software. **Mabel S. Duah:** Data curation, Writing – original draft, Visualization, Investigation. **John Arko-Mensah:** Supervision. **Serwaa A. Bawua:** Writing – review & editing. **Thomas P. Agyekum:** Writing – review & editing. **Julius N. Fobil:** Writing – review & editing.

## Declaration of Competing Interest

The authors declare that they have no known competing financial interests or personal relationships that could have appeared to influence the work reported in this paper.

## Data Availability

No data was used for the research described in the article.

## Appendix Supplementary materials

Image, application 1
