# Supplementary material for: Assessing the combined effect of multiple metal exposures on pregnancy and birth outcomes: Methodological insights in systematic review research
Source: MethodsX. 2024 Jan 6;12:102558. doi: 10.1016/j.mex.2024.102558 (PMC10825682; doi:10.1016/j.mex.2024.102558)
Supplement: Supplementary file 1 [file mmc1.docx]

**Supplementary material and/or additional information**

Table S1: Full search strategy and results

| **PUBMED** | |
| --- | --- |
| **Search ID** | **Query** |
| #1 | ("heavy metals" OR "metals " OR "pollutant" OR "toxic element" OR "metalloid" OR "arsenic" OR "cadmium" OR "mercury" OR "lead" OR "cobalt" OR "zinc" OR "manganese" OR "iron" OR "nickel" OR "chromium") Filters: Humans, English, Female, from 1998/1/1 - 2023/5/6 = 196,569 |
| #2 | ("Stillbirth" OR "perinatal death" OR "spontaneous abortion" OR "miscarriage" OR "preterm birth" OR "birth weight" OR "low birth weight" OR "small for gestational age" OR "intrauterine growth retardation" OR "birth outcome" OR "adverse birth outcome" OR "adverse pregnancy outcome" OR "pregnancy outcome") Filters: Humans, English, Female, from 1998/1/1 - 2023/5/6 = 115,885 |
| #3 | ("mixture*" OR "multi-pollutant*" OR "multiple metals" OR "multi-metal*" OR "joint effect*" OR "joint association*" OR "joint exposure*" OR "overall effect*" OR "overall association*" OR "overall exposure*" OR "joint and individual" OR "co-pollutant*" OR "co-exposure*" OR "combined metal*" OR "mixed metal*" OR "principal component analysis" OR "exploratory factor analysis" OR "Bayesian Kernel Machine Regression" OR "Least Absolute Shrinkage and Selection Operator" OR "penalized regression*" OR "Weighted Quantile Sum" OR "quantile-based g-computation" OR "quantile g-computation") Filters: Humans, English, Female, from 1998/1/1 - 2023/5/6 = 44, 072 |
| #4 | **#1 AND #2 AND #3** Filters: **Humans, English, Female, from 1998/1/1 - 2023/5/6 = 137** |
| **MEDLINE via EBSCOhost** | |
| **Search** | **Query** |
| #1 | (“heavy metals” OR “metals “ OR “pollutant” OR “toxic element” OR “metalloid” OR “arsenic” OR “cadmium” OR “mercury” OR “lead” OR “cobalt” OR “zinc” OR “manganese” OR “iron” OR “nickel” OR “chromium”) = 281, 516 |
| #2 | ("Stillbirth" OR "perinatal death" OR "spontaneous abortion" OR "miscarriage" OR "preterm birth" OR "birth weight" OR "low birth weight" OR "small for gestational age" OR "intrauterine growth retardation" OR "birth outcome" OR "adverse birth outcome" OR "adverse pregnancy outcome" OR "pregnancy outcome") = 178,612 |
| #3 | ("mixture*" OR "multi-pollutant*" OR "multiple metals" OR "multi-metal*" OR "joint effect*" OR "joint association*" OR "joint exposure*" OR "overall effect*" OR "overall association*" OR "overall exposure*" OR "joint and individual" OR "co-pollutant*" OR "co-exposure*" OR "combined metal*" OR "mixed metal*" OR "principal component analysis" OR "exploratory factor analysis" OR "Bayesian Kernel Machine Regression" OR "Least Absolute Shrinkage and Selection Operator" OR "penalized regression*" OR "Weighted Quantile Sum" OR "quantile-based g-computation" OR "quantile g-computation") = 66,486 |
| #4 | #1 AND #2 AND #3 = 284 NB: exact duplicates removed from results |
| Limiters | English Language  Academic/Peer reviewed Journals  1998-2023  Females |
| **SCOPUS** | |
| #1 | (“heavy metals” OR “metals “ OR “pollutant” OR “toxic element” OR “metalloid” OR “arsenic” OR “cadmium” OR “mercury” OR “lead” OR “cobalt” OR “zinc” OR “manganese” OR “iron” OR “nickel” OR “chromium”) = 4,462,517 |
| #2 | ("Stillbirth" OR "perinatal death" OR "spontaneous abortion" OR "miscarriage" OR "preterm birth" OR "birth weight" OR "low birth weight" OR "small for gestational age" OR "intrauterine growth retardation" OR "birth outcome" OR "adverse birth outcome" OR "adverse pregnancy outcome" OR "pregnancy outcome") = 177,299 |
| #3 | ("mixture*" OR "multi-pollutant*" OR "multiple metals" OR "multi-metal*" OR "joint effect*" OR "joint association*" OR "joint exposure*" OR "overall effect*" OR "overall association*" OR "overall exposure*" OR "joint and individual" OR "co-pollutant*" OR "co-exposure*" OR "combined metal*" OR "mixed metal*" OR "principal component analysis" OR "exploratory factor analysis" OR "Bayesian Kernel Machine Regression" OR "Least Absolute Shrinkage and Selection Operator" OR "penalized regression*" OR "Weighted Quantile Sum" OR "quantile-based g-computation" OR "quantile g-computation") = 861,536 |
| Limiters | English Language  Academic/Peer reviewed Journals  1998-2023 |
| #4 | #1 AND #2 AND #3 = 394 |

Table S2: Newcastle-Ottawa quality assessments of the included cohort studies

| **Criteria** | **Selection (4 stars)** | | | | **Comparability of cohorts on the basis of the design or analysis (2 stars)** | | **Outcome assessment (3 stars)** | | | **Total quality score** |
| --- | --- | --- | --- | --- | --- | --- | --- | --- | --- | --- |
| **Sources** | Representativeness of the exposed cohort | Selection of the non-exposed cohort | Ascertainment of exposure | Demonstration that outcome of interest was not present at start of study | Study controls for maternal age | Study controls for any additional factor | Assessment of outcome | Was follow-up long enough for outcomes to occur | Adequacy of follow up of cohorts |  |
| Ashrap et al. (2020) | * | - | * | * | * | * | * | * | * | 8/9 |
| Ashrap et al. (2021) | * | - | * | * | * | * | * | * | * | 8/9 |
| Chen et al. (2021) | * | - | * | * | * | * | * | * | - | 7/9 |
| Deyssenroth et al. (2018) | * | - | * | * | * | * | * | * | - | 7/9 |
| Howe et al. (2021) | * | - | * | * | * | * | * | * | - | 7/9 |
| Howe et al. (2020) | * | - | * | * | * | * | * | * | - | 7/9 |
| Howe et al. (2022) | * | - | * | * | * | * | * | * | - | 7/9 |
| J. M. Y. Hu et al. (2021) | * | - | * | * | * | * | * | * | - | 7/9 |
| Huang et al. (2021) | * | - | * | * | * | * | * | * | * | 8/9 |
| Kim, Meeker, et al. (2020) | * | - | * | * | * | * | * | * | - | 7/9 |
| M.-S. Lee et al. (2021) | * | - | * | * | * | * | * | * | * | 8/9 |
| S. Lee et al. (2020) | * | - | * | * | * | * | * | * | * | 8/9 |

Table S2 Cont: Newcastle-Ottawa quality assessments of the included cohort studies

| **Criteria** | **Selection (4 stars)** | | | | **Comparability of cohorts on the basis of the design or analysis (2 stars)** | | **Outcome assessment (3 stars)** | | | **Total quality score** |
| --- | --- | --- | --- | --- | --- | --- | --- | --- | --- | --- |
| **Sources** | Representativeness of the exposed cohort | Selection of the non-exposed cohort | Ascertainment of exposure | Demonstration that outcome of interest was not present at start of study | Study controls for maternal age | Study controls for any additional factor | Assessment of outcome | Was follow-up long enough for outcomes to occur | Adequacy of follow up of cohorts |  |
| Dou et al. (2022) | * | - | * | * | * | * | * | * | * | 8/9 |
| J. Hu et al. (2022) | * | - | * | * | * | * | * | * | * | 8/9 |
| Wu et al. (2023) | * | - | * | * | * | * | * | * | * | 8/9 |
| L. Zhao et al. (2023) | * | - | * | * | * | * | * | * | * | 8/9 |

Table S2 cont. Newcastle-Ottawa quality assessments of the included cohort studies

| **Criteria** | **Selection (4 stars)** | | | | **Comparability of cohorts on the basis of the design or analysis (2 stars)** | | **Outcome assessment (3 stars)** | | | **Total quality score** |
| --- | --- | --- | --- | --- | --- | --- | --- | --- | --- | --- |
| **Sources** | Representativeness of the exposed cohort | Selection of the non-exposed cohort | Ascertainment of exposure | Demonstration that outcome of interest was not present at start of study | Study controls for maternal age | Study controls for any additional factor | Assessment of outcome | Was follow-up long enough for outcomes to occur | Adequacy of follow up of cohorts |  |
| Rahman et al. (2021) | * | - | * | * | * | * | * | * | - | 7/9 |
| Signes-Pastor et al. (2019) | * | - | * | * | * | * | * | * | - | 7/9 |
| Takatani et al. (2022) | * | - | * | * | * | * | * | * | - | 7/9 |
| Yang et al. (2020) | * | - | * | * | * | * | * | * | * | 8/9 |
| Zhang et al. (2022) | * | - | * | * | * | * | * | * | - | 7/9 |
| H. Zhao et al. (2020) | * | - | * | * | * | * | * | * | * | 8/9 |
| Zilversmit Pao, Harville, Wickliffe, Shankar, and Buekens (2019) | * | - | * | * | * | * | * | * | - | 7/9 |

Table S3: Newcastle-Ottawa quality assessments of the included case-control studies

| **Criteria** | **Selection (4 stars)** | | | | **Comparability of cohorts on the basis of the design or analysis (2 stars)** | | **Exposure assessment (3 stars)** | | | **Total quality score** |
| --- | --- | --- | --- | --- | --- | --- | --- | --- | --- | --- |
| **Sources** | Is the case definition adequate? | Representativeness of the cases | Selection of Controls | Definition of Controls | Study controls for maternal age | Study controls for any additional factor | Ascertainment of exposure | Same method of ascertainment for cases and controls | Non-Response rate |  |
| Hou et al. (2019) | * | * | * | * | * | * | * | * | - | 8/9 |
| Kim et al. (2018) | * | * | * | * | * | * | * | * | - | 8/9 |
| Liu et al. (2022) | * | * | * | * | * | * | * | * | - | 8/9 |
| Ren et al. (2022) | * | * | * | * | * | * | * | * | - | 8/9 |
| Wang et al. (2022) | * | * | * | * | * | * | * | * | - | 8/9 |
| Xu et al. (2022) | * | * | * | * | * | * | * | * | - | 8/9 |

Table S4: AXIS Appraisal tool for Cross-Sectional Studies

| Criteria | Kao, Chien, Fan, Lee, and Jiang (2023) | | | Kim, Xu, et al. (2020) | | | Lazarevic et al. (2022) | | | Lu et al. (2022) | | | Michael et al. (2022) | | |
| --- | --- | --- | --- | --- | --- | --- | --- | --- | --- | --- | --- | --- | --- | --- | --- |
|  | Yes | No | CD/NR | Yes | No | CD/NR | Yes | No | CD/NR | Yes | No | CD/NR | Yes | No | CD/NR |
| 1. Were the aims/objectives of the study clear? | ✔ |  |  | ✔ |  |  | ✔ |  |  | ✔ |  |  | ✔ |  |  |
| 2. Was the study design appropriate for the stated aim(s)? |  | ✔ |  |  | ✔ |  |  | ✔ |  | ✔ |  |  |  | ✔ |  |
| 3. Was the sample size justified? |  | ✔ |  |  | ✔ |  |  | ✔ |  |  | ✔ |  |  | ✔ |  |
| 4. Was the target/reference population clearly defined? (Is it clear who the research was about?) | ✔ |  |  | ✔ |  |  | ✔ |  |  | ✔ |  |  | ✔ |  |  |
| 5. Was the sample frame taken from an appropriate population base so that it closely represented the target/reference population under investigation? | ✔ |  |  | ✔ |  |  | ✔ |  |  | ✔ |  |  | ✔ |  |  |
| 6. Was the selection process likely to select subjects/participants that were representative of the target/reference population under investigation? | ✔ |  |  | ✔ |  |  | ✔ |  |  | ✔ |  |  | ✔ |  |  |
| 7. Were measures undertaken to address and categorise non-responders? |  | ✔ |  |  | ✔ |  |  | ✔ |  |  | ✔ |  |  | ✔ |  |
| 8. Were the risk factor and outcome variables measured appropriate to the aims of the study? | ✔ |  |  | ✔ |  |  | ✔ |  |  | ✔ |  |  | ✔ |  |  |
| 9. Were the risk factor and outcome variables measured correctly using instruments/ measurements that had been trialled, piloted or published previously? | ✔ |  |  | ✔ |  |  | ✔ |  |  | ✔ |  |  | ✔ |  |  |
| 10. Is it clear what was used to determined statistical significance and/or precision estimates? (eg, p values, CIs) | ✔ |  |  | ✔ |  |  | ✔ |  |  | ✔ |  |  | ✔ |  |  |
| 11. Were the methods (including statistical methods) sufficiently described to enable them to be repeated? | ✔ |  |  | ✔ |  |  | ✔ |  |  | ✔ |  |  | ✔ |  |  |
| 12. Were the basic data adequately described? | ✔ |  |  | ✔ |  |  | ✔ |  |  | ✔ |  |  | ✔ |  |  |
| 13. Does the response rate raise concerns about non-response bias? |  |  | ✔ |  |  | ✔ |  |  | ✔ |  |  | ✔ |  |  | ✔ |
| 14. If appropriate, was information about non-responders described? |  | ✔ |  |  | ✔ |  |  | ✔ |  |  |  | ✔ |  |  | ✔ |
| 15. Were the results internally consistent? |  |  | ✔ |  |  | ✔ |  |  | ✔ |  |  | ✔ |  |  | ✔ |
| 16. Were the results for the analyses described in the methods, presented? | ✔ |  |  | ✔ |  |  | ✔ |  |  | ✔ |  |  | ✔ |  |  |
| 17. Were the authors’ discussions and conclusions justified by the results? | ✔ |  |  | ✔ |  |  | ✔ |  |  | ✔ |  |  | ✔ |  |  |
| 18. Were the limitations of the study discussed? | ✔ |  |  | ✔ |  |  | ✔ |  |  | ✔ |  |  | ✔ |  |  |
| 19. Were there any funding sources or conflicts of interest that may affect the authors’ interpretation of the results? |  | ✔ |  |  | ✔ |  |  | ✔ |  |  | ✔ |  |  | ✔ |  |
| 20. Was ethical approval or consent of participants attained? | ✔ |  |  | ✔ |  |  | ✔ |  |  | ✔ |  |  | ✔ |  |  |
| **Overall rating** | 14/20 | | | 15/20 | | | 14/20 | | | 14/20 | | | 13/20 | | |

CD: Cannot determine; NR: Not reported
